# Pituitary-Gonadal and Pituitary–Thyroid Axis Hormone Concentrations before and during a Hypoglycemic Clamp after Sleep Deprivation in Healthy Men

**DOI:** 10.1371/journal.pone.0054209

**Published:** 2013-01-10

**Authors:** Kamila Jauch-Chara, Sebastian M. Schmid, Manfred Hallschmid, Kerstin M. Oltmanns, Bernd Schultes

**Affiliations:** 1 Department of Psychiatry and Psychotherapy, University of Luebeck, Luebeck, Germany; 2 Department of Internal Medicine I, University of Luebeck, Luebeck, Germany; 3 Department of Neuroendocrinology, University of Luebeck, Luebeck, Germany; 4 Interdisciplinary Obesity Center, eSwiss Medical and Surgical Center, St. Gallen, Switzerland; Max Planck Institute for Evolutionary Anthropology, Germany, Germany

## Abstract

Total sleep deprivation (TSD) exerts strong modulatory effects on the secretory activity of endocrine systems that might be related to TSD-induced challenges of cerebral glucose metabolism. Here, we investigate whether TSD affects the course of male pituitary-gonadal and pituitary-thyroid axis related hormones during a subsequent 240-min hypoglycemic clamp. Ten healthy men were tested on 2 different conditions, TSD and 7-hour regular sleep. Circulating concentrations of total testosterone, prolactin (PRL), thyroid stimulating hormone (TSH), free triiodothyronine (fT3), and free thyroxin (fT4) were measured during baseline and a subsequent hypoglycemic clamp taking place in the morning. Basal, i.e. at 07∶00 am measured, concentrations of total testosterone (*P* = 0.05) and PRL (*P*<0.01) were lower while the values of TSH (*P* = 0.02), fT3 (*P* = 0.08), and fT4 (*P* = 0.04) were higher after TSD as compared to regular sleep. During the subsequent hypoglycemic clamp (all measurements from baseline to the end of the clamp analyzed) total testosterone concentrations in the regular sleep (*P*<0.01) but not in the TSD condition (*P* = 0.61) decreased, while PRL levels increased (*P* = 0.05) irrespectively of the experimental condition (*P* = 0.31). TSH concentrations decreased during hypoglycemia (*P*<0.01), with this decrease being more pronounced after TSD (*P* = 0.04). However, at the end of the hypoglycemic clamp concentrations all of the above mentioned hormones did not differ between the two sleep conditions. Our data indicate a profound influence of TSD on male pituitary-gonadal and pituitary-thyroid axis hormones characterized by reduced basal testosterone and PRL levels and increased TSH levels. However, since concentrations of these hormones measured at the end of the 240-min hypoglycemic clamp were not affected by TSD it can be speculated that the influence of TSD on the two endocrine axes is rather short lived or does not interact in an additive manner with their responses to hypoglycemia.

## Introduction

The pituitary-thyroid and pituitary-gonadal axis are important parts of the human endocrine system. Both of these two endocrine axes are crucially involved in the regulation of metabolism, body composition, growth, reproduction, immunity, and psychological well-being [1]–[5]. Physiologically, the functionality, i.e. the secretory activity, of both axes is controlled via negative feedback loops.

Within the pituitary-thyroid axis, the anterior pituitary gland secretes thyrotropin/thyroid stimulating hormone (TSH) which stimulates the thyroid gland to synthesise and to release triiodothyronine (T3) and thyroxine (T4). Free, i.e. unbound and biological active, T3 and T4 (free T3 and free T4, i.e. fT3 and fT4, respectively) inhibit TSH secretion from the anterior pituitary gland directly or via inhibition of hypothalamic TSH-releasing hormone (TRH) release thereby establishing a negative feedback loop [6].

Within the pituitary-gonadal axis, hypothalamic secretion of the gonadotropin-releasing hormone (GnRH) stimulates the release of luteinizing hormone (LH) and follicle-stimulating hormone (FSH) from the pituitary to the blood stream. In men, LH and FSH act on testicular Leydig and Sertoli cells by stimulating testosterone secretion and spermatogenesis, respectively. According to a negative feedback loop testosterone, in turn, inhibits the release of GnRH from the hypothalamus as well as of LH and FSH from the pituitary [7]. Of note, the biological activity of testosterone is modulated by the sex hormone-binding globulin (SHGB) which is released from the liver. High concentrations of SHGB reduce of free fraction of circulating testosterone thereby reducing the biological activity of the hormone [8].

Prolactin (PRL), a hormone which is released from the pituitary, is another important modulator of the pituitary-gonadal function acting at different levels of the axis. High PRL levels inhibit hypothalamic GnRH secretion and the responsiveness of the testicular Leydig cells to LH thereby reducing circulating testosterone concentration [9]–[11].

Beside these complex feedback loops sleep and sleep loss, respectively, also profoundly affects the secretory activity of the pituitary-gonadal and pituitary-thyroid axes. A recent study has demonstrated a marked reduction in circulating testosterone concentrations in young healthy men after 8 consecutive days of sleep time restriction to 5 hours per day [12]. Fittingly, circulating concentrations of testosterone, LH, and PRL are reduced after 24 to 48 hours of total sleep deprivation (TSD) [13]–[15]. Regarding thyroid function, TSD of one night increases TSH concentrations [16], [17] while prolonged total or partial sleep deprivation of several days leads to a reduction of TSH concentrations that is most likely a negative feedback consequence of rising peripheral thyroid hormones [16], [17]. However, the underlying mechanisms of these neuroendocrine changes provoked by TSD are largely unknown.

It might be that sleep loss exerts its influence on neuroendocrine function by affecting central nervous glucose metabolism. Cerebral glucose stores in form of astrocytic glycogen, that are assumed to be replenished during sleep [18], [19], progressively deplete during prolonged wakening [20], [21] which may establish a state of latent central nervous energy deficiency. Thus, it is tempting to speculate that changes in neuroendocrine secretory activity after TSD represent an adaptive response to the TSD-associated challenge of cerebral energy homeostasis.

Hypoglycemia is another condition known to severely affect central nervous energy homeostasis by inducing acute central nervous energy deficiency [22], [23]. Moreover, hypoglycemia provokes profound changes in male pituitary-gonadal and pituitary-thyroid axis activity reflected by a rapid reduction in circulating TSH [24] as well as LH and testosterone [25], [26] concentrations.

Here we questioned how the effects of TSD and hypoglycemia on gonadal and thyroid function impact each other. We hypothesised that TSD potentiates the effects of acute hypoglycemia on these two endocrine axes, which would be reflected by a greater decrease in circulating TSH, fT3, fT4, LH, and testosterone levels during a hypoglycemic clamp experiment following a night on TSD. If this hypothesis was confirmed this would support the assumption that the effects of sleep loss on pituitary-gonadal and pituitary-thyroid axis activity represent a consequence of an altered central nervous energy status.

To test this hypothesis, we exposed 10 healthy men to a stepwise-hypoglycemic clamp after a night of regular 7-hour sleep and after a night of TSD, respectively. Hormonal measures reflecting pituitary-gonadal and pituitary-thyroid secretory activity were assessed during a 30-min baseline period and in 30-min intervals during a subsequent 240-min hypoglycemic clamp procedure.

## Materials and Methods

A total of ten normal weight men (BMI range: 20.7 – 25.0 kg/m^2^) aged 20 – 40 years (mean ± SEM: 25.3±1.4 years) participated in the experiments. Exclusion criteria were shift work, sleep disturbances, acute or chronic illness, neurological or psychiatric diseases, alcohol or drug abuse, smoking, and any kind of current medication. The study was carried out in accordance with the Declaration of Helsinki (2000) of the World Medical Association and was approved by the ethics committee of the University of Luebeck. All participants gave written informed consent prior to participation.

Twelve volunteers were recruited via advertisements, announcements as well as flyers from the local community. Before participation, a structured questionnaire was applied to all volunteers to assess self-reported sleep quality and habitual sleep time. Volunteers with self-reported disturbances in sleep continuity, average sleep duration less than 7 hours per night as well as shift-workers were excluded from the study. Included volunteers reported a habitual sleep time between 7 and 8 hours (on average 459±15.27 min), regarded themselves as ‘good sleepers’ with no difficulties in sleep maintenance, and described their regular sleep as restorative. Also, all participants reported to have had regular sleep-wake cycle during the four weeks before the experiments. However, we did not perform a technology- or diary-based sleep monitoring which leaves the assessment of the sleeping habits of our participants somewhat unobjective.

Following an adaptation night in our laboratory, which include the placement of electrodes for standard polysomnography, each participant was tested on two experimental conditions. In one condition, lights were turned off at 10∶30 pm and volunteers were awakened after on average 422±26 min of sleep. (‘regular sleep’). In the other night (‘TSD’) subjects stayed awake, read and watched movies in a sitting position monitored by the investigator throughout the night. The two experimental nights were spaced at least 2 weeks apart and the order of the experimental conditions was balanced across participants with half of the participants undergoing the TSD night first and the other half the regular sleep night first. Of note, participants were not allowed to take naps during the day before each of the two experimental nights. Two men terminated the study after undergoing only one single experimental condition and thus, were excluded from analyses.

The stepwise-hypoglycemic clamp [24], [27], [28] was started at 7∶30 am after a 30-min baseline period by administration of an insulin bolus of 0.01 IU per kg body weight (BW) human insulin (Insuman Rapid, Aventis, Strasbourg, France) over 2 min. Thereafter, insulin was infused at a constant rate of 1.8 mIU per kg BW per min until the end of the clamp. To control the temporal dynamics of blood glucose reduction, i.e. in order to maintain of the scheduled blood glucose plateaus (4.2, 3.6, 3.1, and 2.5 mmol/l, respectively) for 30 min each a 20% dextrose solution was simultaneously infused at a variable rate. At the end of the last plateau, the insulin infusion was stopped and blood glucose levels were normalized by dextrose infusion. Results on blood glucose, insulin, and classical counterregulatory hormones concentrations obtained during this experiment have been previously reported [28].

Blood samples were collected every 30 minutes, i.e. at the beginning and the end of the baseline as well as of each hypoglycemic plateau. Overall, a total of ten blood samples were taken from each participant at each experimental condition and immediately centrifuged. Thereafter, the obtained serum and plasma were transferred as 600 µl aliquots into Eppendorf tubes (Eppendorf AG, Hamburg, Germany) and frozen at –82°C until hormone determination, which took place in 2007. All serum and plasma samples underwent only one freeze-thaw cycle. Due to the lack of sufficient sample volumes the complete set of 10 measurements on testosterone concentration could be performed only in 8 of the 10 particpants and on LH and PRL in 9 participants.

Circulating concentrations of TSH, fT3, fT4, SHGB, total testosterone, FSH, PRL, and LH were measured in dublicate by an enzyme linked immunoassay system (Immulite, Diagnostic Products Corporation, Los Angeles, USA). Detailed information on the used kits as well as the inter- and intra-assay coefficient of variation (CV) for each hormone are provided in Table 1.

**Table 1:** Characteristics of assays used.

Hormone	Kit	inter-assay CV	intra-assay CV
**thyroid stimulating hormone**	LKTB	<10.0%	<6.2%
**free triiodothyronine**	LKF3	<14.6%	<3.5%
**free thyroxin**	LKFT4	<14.0%	<5.2%
**total testosterone**	LKTW	<6.8%	<7.8%
**luteinizing hormone**	LKLH	<5.0%	<3.5%,
**prolactin**	LKPR	<6.4%	<5.7%
**sex hormone-binding globulin**	LKSH	<6.1%	<6.7%
**follicle-stimulating hormone**	LKFS	<6.3%	<4.6%

All hormones were measured by immunoassay analyzer Immulite, Diagnostic Products Corporation, Los Angeles, USA.

Data were analysed by using the SPSS 20 (IBM SPSS Statistics 20) software. All values are presented as means ± standard deviation (SD). To test for the criteria required for multivariate analyses, i.e. normal distribution, data on each variable were subjected to a Kolmogorov-Smirnov as well as Shapiro-Wilk test. Both tests indicated a normal distribution of all analysed variables. To test whether the assumption of values sphericity was met Mauchlýs test of sphericity was applied which confirmed sphericity of all variables. Statistical analyses were based on analysis of variance (ANOVA) for repeated measures models, including the factor ‘condition’ (for regular sleep vs. TSD) and the factor ‘time’ (for repeated measurements during the baseline period as well as during the stepwise hypoglycemic clamp experiment). Hormonal data obtained at all of the ten blood sampling points were included in the respective ANOVA models. The interaction of the factors ‘sleep’ and ‘time’ was termed ‘sleep by time’ and indicates differential changes in variables across the time depending on the experimental condition, i.e. sleep vs. TSD. Whenever necessary, degrees of freedom were adjusted according to the Greenhouse-Geisser procedure. Measurements from blood samples taken at 07∶00 am (first blood sample) were defined as baseline values. For pairwise comparisons of hormone concentrations at a specific time point during the experiment between the two conditions Student’s t-tests were used. A *P*-value <0.05 was considered to be statistically significant.

The sample size of the study was based on a statistical power calculation with the assumptions made within this calculation deriving from data from foregoing studies (for sleep effect: e.g. [12]–[17], for hypoglycemia effect: e.g. [24], [25]). Here a medium effect size for the effect of ‘sleep’ of >1.16 and of >1.21 for the ‘time/hypoglycemia’ effect for all measured hormones according to Cohen (1988) was considered relevant for the study. Under these assumptions, a total of at least 10 subjects is sufficient to detect medium sized within-subjects effects with a probability of 1−β >80%, which is consider adequate in most studies. Due to the drop out of participants we additionally performed post hoc statistical power calculations which indicated that a total of 10 and, in case of LH and PRL of 9, and in case of testosterone of 8 subjects was sufficient to detect medium sized within-subjects interaction effects with a probability of 1−β >80%, of 1−ß >90%, and 1−ß = 72%, respectively.

## Results

Blood glucose and serum insulin concentrations did not differ between conditions during the baseline period and the hypoglycemic clamp (for details see [28]).

**Figure 1:**
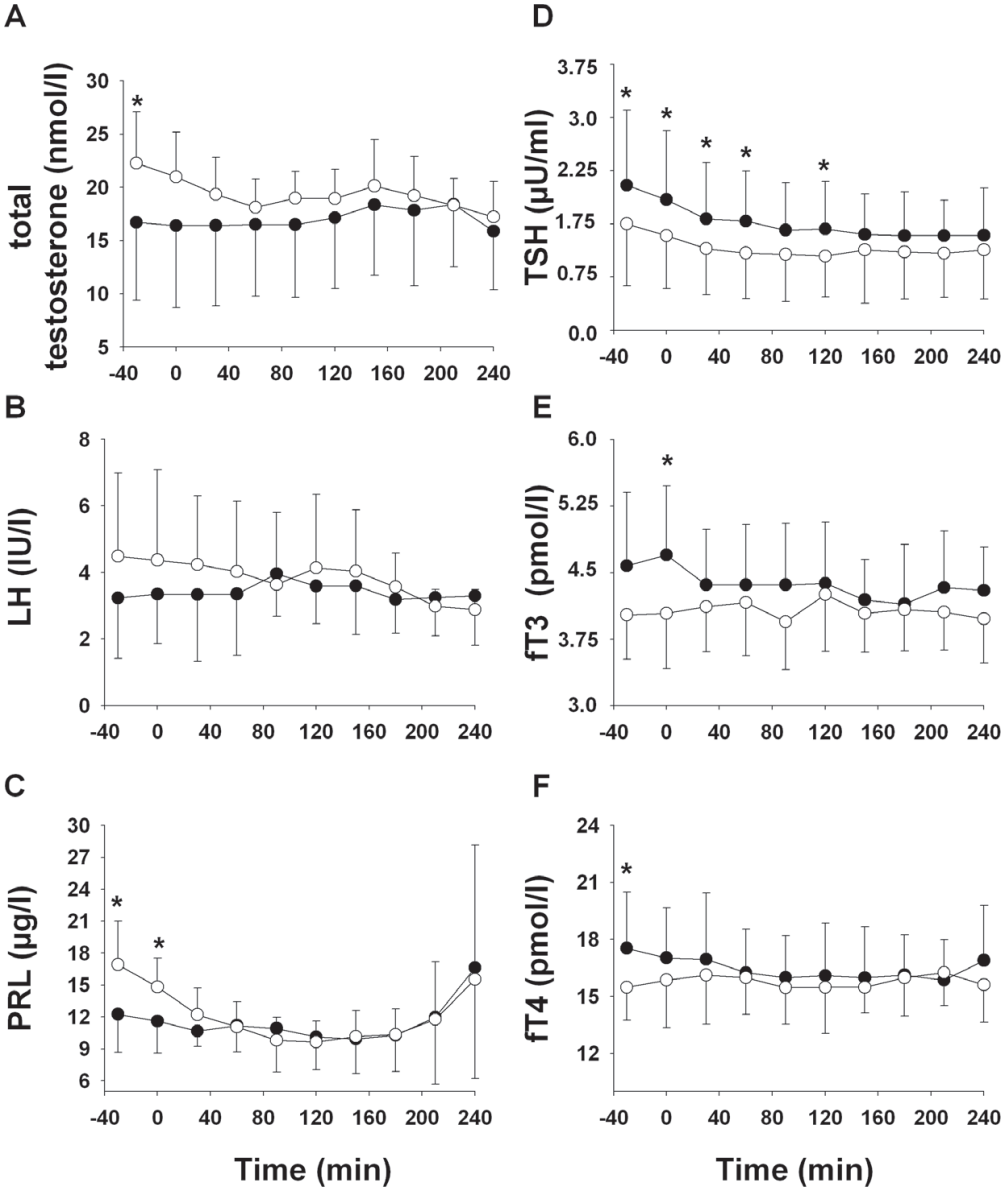
Mean ± SD concentrations of total testosterone (A; n = 8), luteinizing hormone (LH; B; n = 9), prolactin (PRL; C; n = 9), thyroid stimulating hormone (TSH; D; n = 10), free triiodothyronine (fT3; E; n = 10), and free thyroxin (fT4; F; n = 10) during a 30-minute baseline period and the subsequent 240-minute stepwisehypoglycemic clamp after a night of TSD (black circles) and regular sleep (white circles). *P* values derive from Student’s t-test for pairwise comparisons. *P<0.05.

At 07∶00 am, serum testosterone concentrations were significantly lower after TSD than regular sleep (17.3±3.85 vs. 22.2±4.84 nmol/l; t(7) = −2.22, *P* = 0.05), while there were no significant differences in LH levels (3.23±1.82 vs. 4.49±2.50 IU/l; t(8) = −1.10, *P* = 0.30). During the hypoglycemic clamp serum testosterone concentrations progressively decreased in the regular sleep condition, while they remained at their relatively reduced baseline level in the TSD condition (F(4.148,37.335) = 2.44; *P* = 0.01 for the sleep by time interaction term; Figure 1A). Values at the end of the clamp were comparable between conditions (17.2±3.34 vs. 16.8±4.53 nmol/l; t(7) = 1.07, *P* = 0.31). Serum LH levels did not show significant different course between the two sleep conditions across the hypoglycemic clamp (F(2.339,18.711) = 0.97, *P* = 0.42 for sleep by time interaction term; Figure 1B). Serum FSH and SHGB levels did not differ between conditions at any time point (*P*>0.1 for all comparisons, data not shown). Baseline serum PRL levels were lower after TSD than after regular sleep condition (12.3±3.6 vs. 16.9±4.1 µg/l; t(8) = −3.41, *P*<0.01). During hypoglycemia, they rose to comparable peak levels (16.6±10.4 vs. 15.5±12.6 µg/l; t(8) = 0.23, *P* = 0.84; F(1.190,10.714) = 1.21, *P* = 0.31 for the sleep by time interaction term; Figure 1C).

Baseline serum TSH concentrations (2.05±1.06 vs. 1.50±0.87 µU/ml; t(9) = 2.91, *P* = 0.02) were higher and also fT3 concentrations (4.58±0.83 vs. 4.02±0.50 pmol/l; t(9) = 1.99, *P* = 0.08) tended to be higher after TSD than after regular sleep. Accordingly, during the hypoglycemic clamp TSH levels showed a greater decrease in the TSD than regular sleep condition (F(1.395,12.553) = 10.76, *P* = 0.04 for sleep by time interaction term), resulting in similar values at the end of the clamp (1.34±0.67 vs. 1.14±0.70 pmol/l; t(9) = 1.69, *P* = 0.13; Figure 1D). Analyses of serum fT3 levels across the hypoglycemic clamp revealed no significant results (F(1.000,9.000) = 3.52, *P* = 0.09 for the sleep main effect; F(5.004,45.033) = 1.670, *P* = 0.79 for time main effect; F(3.956,35.608) = 1.354, *P* = 0.56 for the sleep by time interaction term; Figure 1E). In comparison to regular sleep, fT4 concentrations were lower after TSD during baseline (17.5±3.0 vs. 15.5±1.7 pmol/l; t(9) = 2.358, *P* = 0.04). During the hypoglycemic clamp there was no significantly changes in fT4 concentrations (F(1.000,9.000) = 1.609, *P* = 0.24 for sleep main effect; F(4.310, 38.791) = 1.59, *P* = 0.19 for time main effect; F(4.041,36.371) = 1.627, *P* = 0.19 for sleep by time interaction term; Figure 1F).

## Discussion and Conclusions

In line with previous results [12]–[17] present data indicate a down-regulation of male pituitary-gonadal and an up-regulation of pituitary-thyroid secretory activity after short-term TSD as reflected by reduced serum concentrations of testosterone as well as increased TSH levels. As previously observed [24], [25], we found a reduction in circulating testosterone and TSH levels during a 240-min hypoglycemic clamp starting in the morning which may reflect an inhibitory influence of hypoglycemia on the secretory activity of both of the two endocrine axes. However, hormonal levels reached similar levels at the end of the hypoglycemic clamp independent of proceeding nocturnal sleep or wakefulness. This finding suggests that the effects of TSD on male gonadal and thyroid function may be rather short-lived or do not interact with their responses to hypoglycemia.

Baseline levels of testosterone were reduced after TSD indicating an inhibitory influence of sleep loss on male gonadal function [1]–[3]. As expected [25], [26], during the hypoglycemic clamp testosterone levels decreased in the regular sleep condition but there was no amplification of the baseline reduction in testosterone levels induced by sleep loss per se in the TSD condition. Although due to the difference in baseline levels the temporal dynamics differed between conditions, this pattern suggests that TSD does not sensitize the male pituitary-gonadal axis to the previously documented [25], [26] suppressive influence of hypoglycemia. This outcome argues against the assumption that the effects of hypoglycemia and sleep loss on pituitary-gonadal secretory activity both rely on alterations of central nervous glucose metabolism as a common mechanism.

Baseline PRL concentrations - in parallel with baseline testosterone - were reduced after the TSD night which is in agreement with previous findings after prolonged sleep deprivation [13]–[15]. While PRL can, in principle, inhibit hypothalamic GnRH secretion and the responsiveness of the testicular Leydig cells to LH [9]–[11] the found reduction of circulating PRL concentrations clearly speak against a role of hormone in mediating the suppressive influence of TSD on testosterone secretion. Instead TSD appears to exert an inhibitory effect on the basal secretion of both PRL and testosterone which in our study did, however, not affected the distinct changes of the two hormones during the subsequent hypoglycemic clamp, i.e. an increase in PRL and a decrease in testosterone.

No significant changes after TSD were found in the concentrations of the two gondotropins LH and FSH which may likely be a consequence a too low frequency in blood sampling rates given the pulsatile nature of the secretion of the hormones [29]. More important, however, is the lack of any change in the SHGB concentration after TSD as found in the present study. Considering that SHGB is the most important binding protein of testosterone it is important to note that the reduction in testosterone after TSD is not compensated by a reduction in SHGB. Given this pattern of finding, it is templing to assume that the free and biologically active fraction of testosterone is likewise reduced after TSD, which may have clinical consequences.

Pituitary-thyroid secretory activity was increased after SD whereas during the hypoglycemic clamp respective hormone levels, as previously observed [24], decreased. This finding might be taken as an indicator that different pathways mediate the effects of SD and hypoglycemia not only on gonadal but also on thyroid function.

An important limitation of our study needs to be pointed out, i.e. the lack of an euglycemic control condition. Both the pituitary-gonadal and the pituitary-thyroid axis secretory activity are well known to show a circadian variation characterized by decreasing hormone levels in the morning hours [30], [31]. Also, the time of awakening plays a major role for the secretory activity of the pituitary-gonadal axis [32], [33]. On this background, we cannot differentiate whether the found reduction in testosterone and TSH levels as well as the increase in PRL during the time of the clamp represents a response to hypoglycemia or a consequence of circadian influences. It might also be that different results would have been obtained if the hypoglycemic clamp had been carried out in the afternoon. However, it should also be noted that previous studies [24], [25], in which we performed similar hypoglycemic clamps along with time-paralleled euglycemic control clamps, clearly showed a much stronger reduction in testosterone and TSH during the hypoglycemic than euglycemic condition. These forgoing findings provide support for our present assumption of a suppressive influence of hypoglycemia on the two studied endocrine axes. Other limitations of our study are the relatively small number of subject tested, which clearly limits the statistical power to detect more discrete effects, and the lack of an objective monitoring of our participants’ sleep habits during the course of the study. Thus, our results should be interpreted with caution and need to be confirmed in larger studies.

In conclusion, we demonstrate profound effects of TSD on the secretory activity of the pituitary-gonadal and pituitary-thyroid axes in healthy men. Our finding of comparable hormones levels in the end of the hypoglycemic clamp independent of preceding sleep loss fits with our previous observation that the absolute counterregulatory response to hypoglycemia as assessed by ACTH, cortisol, and catecholamine levels is likewise not affected by previous sleep deprivation [28]. Taken together, these results might indicate that the effect of sleep loss on the pituitary-gonadal and pituitary-thyroid axes is mediated by mechanisms distinct from alterations in central nervous glucose metabolism.
